# History of a model plant growth-promoting rhizobacterium, *Bacillus velezensis* GB03: from isolation to commercialization

**DOI:** 10.3389/fpls.2023.1279896

**Published:** 2023-10-11

**Authors:** Seonghan Jang, Soo-Keun Choi, Huiming Zhang, Shouan Zhang, Choong-Min Ryu, Joseph W. Kloepper

**Affiliations:** ^1^ Infectious Disease Research Center, Research Institute of Bioscience and Biotechnology (KRIBB), Yuseong-gu, Daejeon, Republic of Korea; ^2^ Department of Biosystems and Bioengineering, KRIBB School of Biotechnology, Korea University of Science and Technology (UST), Yuseong-gu, Daejeon, Republic of Korea; ^3^ Shanghai Center for Plant Stress Biology, Center for Excellence in Molecular Plant Sciences, Chinese Academy of Sciences, Shanghai, China; ^4^ Tropical Research and Education Center, Department of Plant Pathology, University of Florida-IFAS, Homestead, FL, United States; ^5^ Department of Entomology and Plant Pathology, Auburn University, Auburn, AL, United States

**Keywords:** PGPR, antimicrobial peptides, induced systemic resistance, induced systemic tolerance, nonribosomal peptide synthetases, microbiome

## Abstract

*Bacillus velezensis* strain GB03 is a Gram-positive rhizosphere bacterium known for its ability to promote plant growth and immunity. This review provides a comprehensive overview of the research on GB03 from its initial discovery in Australian wheat fields in 1971 to its current applications. Recognized as a model plant growth-promoting rhizobacterium (PGPR), GB03 has exhibited outstanding performance in enhancing the growth and protection of many crop plants including cucumber, pepper, wheat, barley, soybean, and cotton. Notably, GB03 has been reported to elicit plant immune response, referred to as induced systemic resistance (ISR), against above-ground pathogens and insect pests. Moreover, a pivotal finding in GB03 was the first-ever identification of its bacterial volatile compounds, which are known to boost plant growth and activate ISR. Research conducted over the past five decades has clearly demonstrated the potential of GB03 as an eco-friendly substitute for conventional pesticides and fertilizers. Validating its safety, the U.S. Environmental Protection Agency endorsed GB03 for commercial use as Kodiak^®^ in 1998. Subsequently, other compounds, such as BioYield™, were released as a biological control agent against soil-borne pathogens and as a biofertilizer, utilizing a durable spore formulation. More recently, GB03 has been utilized as a keystone modulator for engineering the rhizosphere microbiome and for eliciting microbe-induced plant volatiles. These extensive studies on GB03 underscore its significant role in sustainable agriculture, positioning it as a safe and environmentally-friendly solution for crop protection.

## 1 Introduction

**Figure 1:**
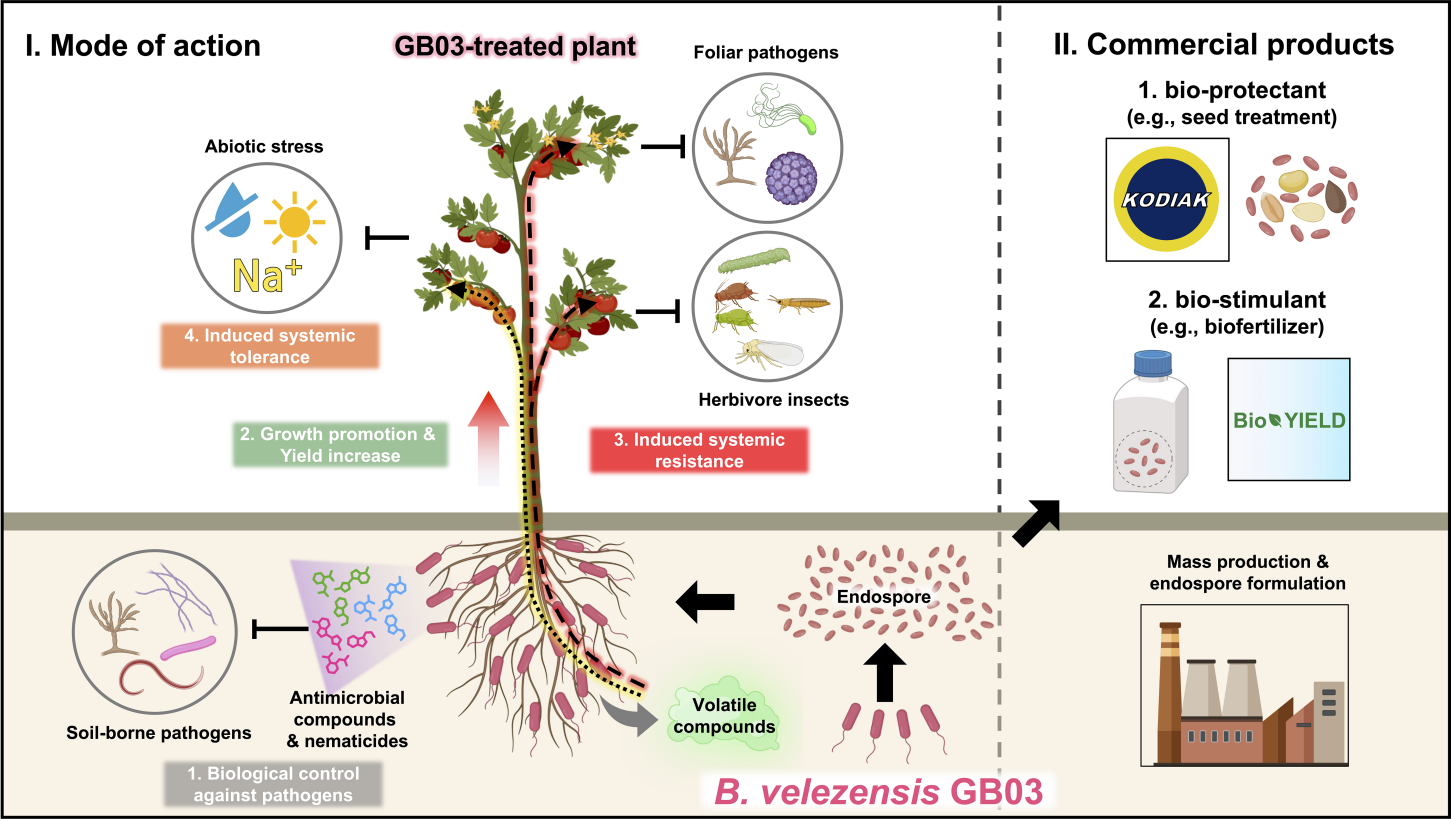
An overview of the plant beneficial effects and commercialization of *Bacillus velezensis* strain GB03. The ‘mode of action’ panel on the left demonstrates that the PGPR *B velezensis* GB03 colonizes the roots of plants. The colonized plants show better growth and higher yield than the non-colonized plants. Upon colonization, GB03 produces a cocktail of antimicrobial substances and nematicides, which inhibit the growth of soil-borne pathogens or nematodes. Additionally, GB03 colonization on plant roots triggers ISR, priming plant immunity to defend against foliar pathogens and insect herbivores. GB03-treated plants also exhibit resistance to abiotic stresses such as drought and high salinity. The ‘commercialized products’ panel on the right displays some of the commercialized products derived from GB03 and their applications in crop production. The endospores of *B velezensis* GB03 have been commercialized as bio-protectants (e.g., Kodiak), which are used to treat plant seeds and provide protection against numerous soil-borne pathogens. Furthermore, the endospores of GB03 have been developed as bio-stimulants (e.g., BioYield), which enhance the yield of diverse crop plants.

A recent paradigm shift, upon revisiting the role of soil microbes, including rhizosphere bacteria (rhizobacteria), in plant health, suggests that the rhizosphere microbiome is a key determinant of plant health (Berendsen et al., 2012). Plant growth-promoting rhizobacteria (PGPR), which colonize plant roots and have beneficial effects on plant growth and immunity, draw special attention due to their protective effects on crops and ecosystems (Kloepper et al., 2004a). Rhizobacteria strains of various genera are designated as PGPR. Fluorescent pseudomonads are particularly intriguing due to their versatile metabolism, rapid growth, and strong mobility (Bakker et al., 2007). *Streptomycetaceae* is a family of exceptional antibiotic producers that can suppress plant pathogens, mostly fungal pathogens. However, major barriers to the agricultural usage of *Streptomycetes* spp. and *Pseudomonas* spp. are difficulties associated with mass production in liquid culture and poor long-term storage because of a short shelf-life, respectively (Emmert and Handelsman, 1999). *Bacillus* spp. and *Paenibacillus* spp. are strong candidates as bacterial species that could overcome the drawbacks of *Streptomycetes* spp. and *Pseudomonas* spp. (Palaniyandi et al., 2013). In addition to their long shelf-life, *Bacillales* spp. produce diverse antimicrobial peptides that suppress the growth and fitness of both human and plant pathogenic microbes (Ongena and Jacques, 2008; Sumi et al., 2015). As mentioned above, the genus *Bacillus* is a strong candidate for commercialization in the fertilizer or microbicide industry. Among the *Bacillus* species, we focus on the PGPR *Bacillus velezensis* strain GB03, a well-studied and representative *Bacillus* strain. It has the ability to protect plants against foliar pathogens, soil-borne pathogens, and abiotic stress. Additionally, GB03 can promote plant growth and increase crop yield (**Figure 1**).

## 2 Isolation and renaming of *Bacillus subtilis* strain A13

*B. subtilis* strain A13 was isolated in 1971 from the lysed mycelium of *Sclerotium rolfsii*, which was found in wheat-field soil in Glen Osmond, South Australia. Since 1989, it has been used as a model PGPR by Kloepper and colleagues because it is moderately competitive in the rhizosphere. (Broadbent et al., 1971; Kloepper et al., 1989). Early investigations of *B. subtilis* strain A13 demonstrated its remarkable capacity to promote the growth of several plant genera in the greenhouse and provide biological control against root pathogens (Broadbent et al., 1971; Broadbent, 1977; Kloepper et al., 1989). For instance, the application of *B. subtilis* strain A13 to the soil decreased damping-off and wire-stem diseases in pepper seedlings caused by *Rhizoctonia solani*. Moreover, A13 was successfully applied in field conditions, and its use on peanut seeds enhanced the overall crop yield (Turner, 1991). Subsequently, in collaboration with Gustafson, a Texas-based seed company, Kloepper and colleagues attempted to develop an A13-based cotton seed treatment through multiple host passages of A13 in cotton plants for selective cultivation (Kloepper et al., 1991). Gustafson renamed strain A13 as Gustafson Biological number 03 (GB03). Further evaluation revealed that *B. subtilis* strain GB03 exerts effective biological control over root pathogens in peanut and cotton (Kloepper et al., 1991; Brannen and Kenney, 1997). Subsequently, as a result of comprehensive taxonomic reclassification within the *Bacillus* genus, *B. subtilis* strain GB03 was redesignated as *B. amyloliquefaciens* strain GB03, which has now been formally renamed as *B. velezensis* strain GB03 (Fan et al., 2017; Mullins et al., 2020). *B. velezensis* strain GB03 continues to serve as a model bacterium for studying plant-bacteria interactions.

## 3 Biological control and plant growth promotion

Several studies have investigated the plant growth-promoting effects of the *B. velezensis* strain GB03 alone or in combination with other PGPR strains. After conducting an extensive study on the biological control capacity and plant growth-promoting effect of GB03 (Broadbent et al., 1971; Turner, 1988; Turner and Backman, 1991), GB03 was commercialized as Kodiak^®^ (Gustafson, Inc, Plano, TX, USA), which has been widely used for seed treatment in various crops, including cotton. This is the first biological control agent based on bacilli against plant pathogens. In cotton, seed treatment with Kodiak^®^ (GB03) and chemical fungicides improves plant stand and suppresses soil-borne pathogens, including *R. solani* and *Fusarium oxysporum* f. sp. *vasinfectum* (Brannen and Kenney, 1997). GB03 produces an antibiotic called iturin, which acts against cotton pathogens. Kodiak^®^ has also been registered for seed treatment in wheat, barley, soybean, cotton, and other agricultural crops to suppress fungal pathogens belonging to the genera *Rhizoctonia, Fusarium, Alternaria*, and *Aspergillus* (Anckaert et al., 2021). This topic is further discussed in section 8: Formulation and commercialization.

### 3.1 Promotion of plant growth

Numerous studies have been conducted on the plant growth-promoting effects of GB03 when used alone or in combination with other PGPR strains (**Table 1**). Kodiak, a biological formulation based on strain GB03, was shown to increase plant growth and promote yield in peanut and cotton in the 1990s (Turner, 1991; Brannen and Backman, 1993; Brannen and Backman, 1994).

**Table 1:** Summary of the plant growth-promoting effects of GB03.

Effect on plants	Treatments	Application method	Plant species	Type of trial	References
Plant growth promotion, yield increase	GB03 (Kodiak)	Seed treatment	Peanut	Field	Turner and Backman, 1991
		Seed treatment	Cotton	Field	Brannen and Backman, 1993; Brannen and Backman, 1994
Increased main runner length	GB03 + *Bacillus pumilus* INR7 + *Curtobacterium flaccumfaciens* ME1	Seed treatment	Cucumber	Field	Raupach and Kloepper, 1998
Increased stem length	GB03 + *Bacillus amyloliquefaciens* IN937a	Seed treatment	Cucumber	Field	Raupach and Kloepper, 2000
Improved transplant vigor and survival	GB03 + one of the other bacilli PGPR	Addition to growth media	Tomato and pepper	Field	Kokalis-Burelle et al., 2002
Increased yield	GB03 + *B. pumilus* INR7		Tomato and pepper		
	GB03 + *Bacillus subtilis* IN937b		Pepper		
Increased shoot length and seedling weight	GB03 + one of the other bacilli PGPR	Addition to growth media	Mustmelon and watermelon	Greenhouse, field	Kokalis-Burelle et al., 2003
Strengthened plant vigor and increased plant height, shoot fresh weight, leaflet number per plant, and leaf surface area	GB03 + one or two bacilli PGPR strains mixed in chitosan	Addition to soilless potting media	Tomato, bell pepper, cucumber, tomato, and tobacco	Greenhouse, field	Kloepper et al., 2004a
Increased plant height, fresh weight, and flower and fruit number	GB03 + one of the other bacilli PGPR strains formulated with chitosan	Addition to soilless potting media	Tomato	Greenhouse	Murphy et al., 2003
Increased root biomass	GB03 + *B. subtilis* FZB24	Seed drench + soil drench	Corn	Greenhouse	Myresiotis et al., 2015
Increased seedling growth	GB03	Addition to growth media	*Arabidopsis*	Growth chamber	Ryu et al., 2003; Xie et al., 2009
Increased branching, shoot and root length, whole-plant fresh and dry weight, and leaf area	GB03	Seed soaking	A herbal plant *Codonopsis pilosula*	Laboratory, greenhouse	Wu et al., 2016
Increased leaf area, node number, and shoot and root biomass	GB03	Root drench	Peppermint	Greenhouse	Del Rosario Cappellari et al., 2015
Increased fresh and dry weight	GB03	Inoculation of MS media	Arugula (*Eruca sativa*)	Growth chamber	Chou, 2013
Increased shoot and root growth	GB03	Inoculation of MS media	Grass (*Puccinellia tenuiflora*)	Laboratory	Niu et al., 2016

#### 3.1.1 Promotion of cucumber growth

In field trials conducted in different years, seed treatment of cucumber with a mixture of GB03 and *B. amyloliquefaciens* IN937a led to a significant increase in plant growth compared to the untreated control (Raupach and Kloepper, 2000). Shoot length was significantly increased in PGPR treatments each year, even without soil fumigation with methyl bromide. In another field trial, it was found that seed treatment with GB03, in combination with *Bacillus pumilus* INR7 and *Curtobacterium flaccumfaciens* ME1, significantly increased the length of the main runner in cucumber compared to the control (Raupach and Kloepper, 1998).

#### 3.1.2 Promotion of tomato and pepper growth

Similarly, Kokalis-Burelle and co-workers reported that the application of biological preparations containing GB03 and another bacilli PGPR strain to transplant (plug) soilless mix resulted in a significant increase in the growth of transplanted tomato and pepper seedlings. This finding was observed in field trials conducted in Sanford, Florida (FL.) USA., using sandy loam soil (Kokalis-Burelle et al., 2002). PGPR treatments significantly improved the vigor and survival of both tomato and pepper plants. A formulation of GB03 and *B. pumilus* INR7 significantly increased the yield of extra-large tomato fruits and the overall yield compared to the untreated control. Pepper yield was enhanced with two formulations containing GB03 (GB03 + *B. subtilis* IN937b; GB03 + *B. pumilus* INR7).

Formulations of GB03, along with one of the other bacilli PGPR strains, in chitosan (as the carrier) promoted plant growth and induced systemic resistance (ISR) against *Cucumber mosaic virus* (CMV) in tomato (Murphy et al., 2003). Treatment of tomato plants with formulations containing two PGPR strains resulted in significantly greater plant height, fresh weight, and the numbers of flowers and fruits compared to the untreated control. GB03, when applied alone or in combination with *B. subtilis* FZB24 (Companion), increased root biomass in corn by 38-65% compared to the uninoculated control (Myresiotis et al., 2015).

In a series of greenhouse and field experiments in Sanford, FL, GB03 was evaluated individually and in combination with *B. amyloliquefaciens* IN937a and *B. subtilis* IN937b. These are PGPR strains with different modes of action, and they were tested in a formulation with chitosan (Kloepper et al., 2004a). PGPR strains and chitosan were applied to the growth media before sowing the seeds to produce transplants. GB03 combined with one or two bacilli PGPR strains in chitosan significantly enhanced various plant growth parameters, including plant vigor, plant height, shoot fresh weight, leaflet number per plant, and leaf surface area, in tomato, bell pepper, cucumber, and tobacco. A bioproduct, BioYield, was then developed by Gustafson, LLC for use on various crops (Kokalis-Burelle et al., 2006). GB03, as a PGPR, transplant amendments and its effects on indigenous rhizosphere microorganisms were further evaluated.

#### 3.1.3 Promotion of growth in other crops

In muskmelon and watermelon, six formulations containing GB03 and other PGPR strains, which were previously shown to increase plant growth in other vegetable transplants, were evaluated in greenhouse and field trials in Alabama (AL) and FL. USA (Kokalis-Burelle et al., 2003). Several treatments containing GB03 and other bacilli PGPR significantly increased the shoot length, shoot weight, and stem diameter (caliper) of muskmelon and watermelon seedlings and transplants in the greenhouse. The PGPR treatments also increased the root weight of muskmelon seedlings.

The effect of GB03 was also evaluated in an herbal crop, *Codonopsis pilosula*. Seed-soaking treatment with GB03 enhanced branching, shoot and root length, whole-plant fresh and dry weight, leaf area, and chlorophyll content (Wu et al., 2016). In subsequent studies, GB03 also increased the transpiration rate, stomatal conductance, and net photosynthetic rate. In greenhouse experiments on peppermint, GB03 increased leaf area, node number, and shoot and root biomass compared to the control (Del Rosario Cappellari et al., 2015). Plants treated with GB03 exhibited higher trichome and stomatal densities, as well as greater monoterpene content, compared to the untreated control plants. In addition, GB03 was found to increase the fresh and dry weight of arugula (*Eruca sativa*), an agricultural salad crop, through the production of volatile organic compounds (VOCs) (Chou, 2013).

In addition to *Arabidopsis* and many horticultural and agricultural crops, GB03 was also reported to promote shoot and root growth in *Puccinellia tenuiflora*, a grass species, under salinity stress (Niu et al., 2016). Treatment with GB03 reduced Na^+^ accumulation in *P. tenuiflora* plants, but had no effect on K^+^ accumulation. Therefore, GB03 enhanced the selective absorption capacity of K^+^ over Na^+^ in the halophyte *P. tenuiflora*.

#### 3.1.4 Promotion of growth in model plant *Arabidopsis*

The mechanisms underlying plant growth promotion by GB03 were extensively studied in *Arabidopsis thaliana* (*Arabidopsis*) in the early 2000s. Ryu et al. (2003) reported that *B. velezensis* GB03 released a mixture of volatile compounds in I-plates, which significantly enhanced the growth of *Arabidopsis* seedlings (Ryu et al., 2003). I-plates are Petri dishes with a vertical plastic divider across the center, which separates the Petri-plate into two halves. This division prevents the spread of soluble compounds in the media. Two volatile compounds, namely 2,3-butanediol and 3-hydroxy-2-butanone (acetoin), were exclusively released by GB03, resulting in the greatest level of growth promotion in *Arabidopsis*. In addition, the application of 2,3-butanediol enhanced plant growth in *Arabidopsis*. However, bacterial mutants that were defective in 2,3-butanediol and acetoin synthesis were unable to promote seedling growth. In another study, GB03 induced sustained growth promotion in *Arabidopsis* and increased seed set after long-term exposure. This was because GB03 volatiles elevated photosynthetic capacity and iron (Fe) accumulation (Xie et al., 2009).

In *Arabidopsis*, GB03 increased photosynthetic capacity by enhancing photosynthetic efficiency and chlorophyll content (Xie et al., 2009). Elevation of sugar accumulation and suppression of classical glucose signaling responses were responsible for the increased photosynthesis. GB03 had no effect on the photosynthetic capacity of *Arabidopsis* mutants defective in hexokinase-dependent sugar signaling. Plants exposed to GB03 volatiles showed a decrease in transcript levels of genes related to abscisic acid (ABA) biosynthesis in leaves as well as a connection between sugar and ABA sensing. Furthermore, the exogenous application of ABA abolished the increase in photosynthetic efficiency and chlorophyll content induced by GB03. This demonstrates that GB03 promotes photosynthesis through the modulation of endogenous sugar/ABA signaling.

### 3.2 Biological control and induced resistance

In addition to promoting plant growth, numerous studies have demonstrated the effectiveness of *B. velezensis* GB03 in biologically controlling phytopathogens. *B. velezensis* GB03 colonizes the plant root system and competes with fungal pathogens in the soil. Since the early 2000s, GB03 has been extensively studied for its efficacy against plant pathogens including fungi, bacteria, viruses, nematodes, and insects (**Table 2**).

**Table 2:** Biocontrol of plant diseases and insects using GB03.

Treatment	Application method	Target Diseases and pathogens	Plant species	Type of trial	Reference
GB03	Seed treatment	Fusarium wilt	Cotton	Greenhouse	Zhang et al., 1996
GB03	Incorporation of cell suspension into soil before planting	Stem canker caused by *Rhizoctonia solani*	Potato	Greenhouse	Brewer and Larkin, 2005
GB03	Seed and soil treatment	Fusarium wilt	Chickpea	Microplot	Landa et al., 2004
GB03 GB03 + IN937a	Soil drench	Fusarium crown and root rot	Tomato	Greenhouse	Myresiotis et al., 2012
GB03 GB03 + one or two other PGPR strains	Seed treatment	Anthracnose, Angular leaf spot, and cucurbit wilt	Cucumber	Greenhouse	Raupach and Kloepper, 1998
GB03 GB03 + one or two other PGPR strains (INR7, ME1)	Seed treatment	Anthracnose, Angular leaf spot	Cucumber	Field	Raupach and Kloepper, 1998; Raupach and Kloepper, 2000
GB03 + one of the other bacilli PGPR	In formulation with chitosan	Gummy stem blight and Angular leaf spot	Watermelon Muskmelon	Field	Kokalis-Burelle et al., 2002; Kokalis-Burelle et al., 2003
GB03 + one of the other bacilli PGPR	In formulation with chitosan	*Pythium*, *Fusarium* and Root-knot nematode	Pepper	Field	Kokalis-Burelle et al., 2002
GB03 + IN937a + IN937b	In formulation with chitosan	Angular leaf spot	Cucumber	Greenhouse, field	Kloepper et al., 2004a
		Bacterial spot	Tomato		
GB03 GB03 + IN937a GB03 + IN937b		Crown and root rot, Root-knot nematode	Tomato	Field	
GB03	Cell suspension drops on wound	Green mold	Citrus	Dish pan	Zhang and Dou, 2002
GB03	Addition to growth media	Soft rot	*Arabidopsis*	Growth chamber	Ryu et al., 2004
		*Botrytis cinerea*			Sharifi and Ryu, 2016
GB03	Foliar spray	Blossom fire blight	Pear	Field	Bahadou et al., 2017
GB03 GB03 + *B. pumilus* T4	Seed treatment	*Bean common mosaic virus*	Cowpea	Screen-house, field	Shankar et al., 2009
GB03 + IN937a	In formulation with chitosan	*Cucumber mosaic virus*	*Arabidopsis*	Greenhouse	Ryu et al., 2007
GB03 + one of the other bacilli PGPR	In formulation with chitosan	*Cucumber mosaic virus*	Tomato	Greenhouse	Murphy et al., 2003
GB03	Inoculated to the non-plant portion of I-plate chamber	Beet armyworm	*Arabidopsis*	I-plates in growth chamber	Aziz et al., 2016
GB03	Seed treatment	Diamondback moth	Arugula	Growth chamber	Dos Santos et al., 2021
GB03	Seed coating + soil drench	Increased root uptake and systemic translocation of ASM	Tomato	Greenhouse	Myresiotis et al., 2014
GB03	Seed drench + soil drench	Increased uptake of thiamethoxam, a new class of neonicotinoid insecticides	Corn	Greenhouse	Myresiotis et al., 2015

#### 3.2.1 Biological control of fungi

Phytopathogenic fungi including the genus *Fusarium*, cause severe diseases in many crops, resulting in significant economic losses. Thus, GB03 has been studied for its ability to control these fungal pathogens. Under greenhouse conditions, seed treatment with GB03 suppressed the incidence and severity of Fusarium wilt in cotton grown in soil infested with *F. oxysporum* f. sp. *vasinfectum* and *Meloidogyne incognita* (Zhang et al., 1996). When applied as a seed treatment, GB03 significantly reduced *Fusarium* colonization in the taproot and secondary roots of cotton seedlings compared to the untreated control.

In the greenhouse, 28 biocontrol strains including GB03 were tested for their ability to suppress the growth of *R. solani* on potatoes. GB03 was one of the strains that effectively reduced the severity of stem canker by 40-49% compared to the untreated controls in all trials (Brewer and Larkin, 2005). The combination of GB03 and a *Trichoderma virens* strain resulted in greater control of stem canker than each strain alone.

In a three-year experiment conducted in field microplots, GB03 applied alone or in combination with non-pathogenic *F. oxysporum* was among the most effective treatments for suppressing Fusarium wilt in chickpea (Landa et al., 2004). Results from this study demonstrated the importance and feasibility of integrating biological control with existing partially effective control practices for the improved management of Fusarium wilt in chickpea.

Four strains of bacilli PGPR, including GB03, were applied individually and in combination to determine their effects on Fusarium crown and root rot, which is caused by *Fusarium oxysporum* f. sp. *radicis-lycopersici* (*Forl*), in tomatoes (Myresiotis et al., 2012). When applied individually, GB03 suppressed the disease more effectively than the other PGPR strains. Through screening combination application with other PGPR strains, GB03 in combination with IN937a provided a higher control of the disease. More effective control was achieved when GB03 and other PGPR strains were applied in combination with either acibenzolar-S-methyl (ASM) or hymexazol.

Raupach and Kloepper reported that in greenhouse trials, GB03 either alone or in combination with one or two other PGPR strains, significantly protected cucumber plants against anthracnose caused by *Colletotrichum orbiculare* (Raupach and Kloepper, 1998). When *C. orbiculare* was challenged with one or two other pathogens, such as *Pseudomonas syringae* pv. *lachrymans*, which cause angular leaf spot, and *Erwinia tracheiphila*, which cause cucurbit wilt, were found to be significantly reduced in severity when GB03 was applied alone or in combination with other PGPR strains in cucumber. In field trials, cucumber plants were spray-inoculated with *C. orbiculare* and *P. syringae* pv. *lachrymans*-infected source plants. GB03 applied in combination with two other PGPR strains (*B. pumilus* INR-7 and *Curtobacterium flaccumfaciens* ME-1) significantly reduced the severity of anthracnose and angular leaf spot. A mixture of three PGPR strains (GB03+ INR7 + ME1), when applied as a seed treatment, resulted in a significant reduction in disease severity, comparable to the levels achieved by applying using Actigard (Bion in Europe and Asia), a synthetic SAR inducer, as a foliar spray.

Field trials were also conducted by Raupach and Kloepper in Shorter, AL to evaluate the effect of GB03, applied alone or in combination with other PGPR strains, on cucumber plants against naturally occurring foliar diseases (Raupach and Kloepper, 2000). Cucumber plants were infested by both *C. orbiculare* and *P. syringae* pv. *lachrymans*. All PGPR treatments, including GB03 alone or in combination with one or two other PGPR strains, significantly reduced the severity of anthracnose and angular leaf spot compared to the untreated control, both with and without methyl bromide. Mixtures of GB03 with other PGPR strains showed improved disease control in field trials, both with and without the use of methyl bromide.

Based on the results of previous studies, GB03 was subsequently evaluated as an important PGPR strain in formulations with other PGPR strains used for vegetable transplant production. In the greenhouse, treatments with PGPR containing GB03 resulted in a significant reduction in gummy stem blight caused by *Didymella bryoniae* in watermelon, compared to the control (Kokalis-Burelle et al., 2003). In Florida, USA, field trials were conducted in sandy loam soil to evaluate pepper and tomato transplants grown in a seedling mix amended with PGPR formulations containing GB03 (Kokalis-Burelle et al., 2002). Pepper root condition was improved with formulations containing GB03, such as LS213, LS256, and LS261, compared to the untreated control. LS213 and LS261 significantly decreased the number of *Pythium* colonies, and LS213 significantly reduced the number of *Fusarium* isolates from pepper roots 45 days after planting, compared to the untreated control. In both greenhouse and field experiments, *B. velezensis* GB03 was evaluated alone or in combination with *B. amyloliquefaciens* IN937a and *B. subtilis* IN937b, which are PGPR strains known to have different modes of action (Kloepper et al., 2004a). The evaluation was conducted using a formulation with chitosan. Treatments with GB03 combined with one of the PGPR strains in chitosan significantly reduced disease incidence in tomato, bell pepper, cucumber, and tobacco. Treatments containing GB03 suppressed crown and root rot on tomato in field trials, both with and without soil fumigation.

In citrus, the application of GB03 as a preventative treatment reduced green mold, which is an economically significant postharvest disease of oranges caused by *Penicillium digitatum*, by 11.1–55.6% compared to the untreated control (Zhang and Dou, 2002). This is important because the effective control of postharvest diseases with GB03 can serve as a valuable alternative to chemical control, which is desired by consumers for food safety.

Sharifi and Ryu conducted a study on GB03 to determine the efficacy of its bacterial volatile compounds (BVCs) in preventing *Botrytis cinerea* infection in *Arabidopsis* (Sharifi and Ryu, 2016). GB03 protected *Arabidopsis* seedlings against the necrotrophic fungus *B. cinerea* by emitting volatile compounds through ISR, rather than through direct antagonism.

In summary, GB03 has consistently demonstrated its effectiveness in controlling a wide range of phytopathogenic fungi across various crops and conditions, both alone and in combination with other PGPR strains. These findings underscore the versatility and potential of GB03 as a biocontrol agent, offering a promising alternative to chemical treatments for sustainable agriculture.

#### 3.2.2 Biological control of phytopathogenic bacteria

Bacterial pathogens cause numerous diseases in crops, resulting in substantial economic losses and raising concerns about food security. Biological control against phytopathogenic bacteria is an important strategy in the management of plant diseases. In addition to fungal pathogens, numerous studies have shown that *B. velezensis* GB03 is also effective in controlling phytopathogenic bacteria. In greenhouse studies, seed treatment with GB03 significantly reduced bacterial foliar diseases on cucumber, such as angular leaf spot caused by *P. syringae* pv. *lachrymans* and cucurbit wilt caused by *E. tracheiphila* (Raupach and Kloepper, 1998). In the first-year field trial in AL. USA., the application of GB03 alone, as well as in combination with one or two other PGPR strains, resulted in a lower severity of angular leaf spot caused by *P. syringae* pv. *lachrymans* after either artificial inoculation or using infected source plants. In the second-year field trial, the combination of GB03, INR7 and ME1 proved to be the most effective treatment for controlling angular leaf spot and anthracnose. This treatment was found to be equivalent to Actigard (active ingredient, acibenzolar-S-methyl), an SAR inducer.

In a two-year field trial in AL, Raupach and Kloepper evaluated the effectiveness of GB03 and other PGPR strains in controlling foliar diseases in cucumber. The trials included angular leaf spot in 1996 and a mixed infestation of angular leaf spot and anthracnose in 1997 (Raupach and Kloepper, 2000). In both years, GB03 significantly reduced the severity of foliar diseases, including angular leaf spot, both with and without fumigation with methyl bromide, compared to the untreated control. In addition, the application of other PGPR strains and GB03 mixtures provided a higher level of protection against angular leaf spot and other foliar diseases in cucumber plants compared to the application of PGPR alone.

In the greenhouse, four PGPR treatments containing GB03 reduced angular leaf spot on watermelon compared to the untreated and formulation carrier controls (Kokalis-Burelle et al., 2003). In muskmelon, one treatment reduced the occurrence of angular leaf spot compared to the untreated and carrier control groups. In another greenhouse study, a mixture of GB03 and a PGPR strain IN937a in chitosan was used to treat the potting mix for transplant production. The treatment significantly reduced bacterial spot (caused by *Xanthomonas axonopodis* pv. *vesicatoria*) on tomatoes and angular leaf spot on cucumbers (Kloepper et al., 2004a). In a field trial conducted in FL. USA., the application of GB03 alone and in combination with either IN937a or IN937b in chitosan significantly reduced bacterial spot disease on tomato leaves and fruits compared to the untreated control.

In a commercial pear orchard in Morocco, GB03 alone reduced blossom fire blight, caused by the bacterial pathogen *Erwinia amylovora*, by 64% compared to the untreated control (Bahadou et al., 2017). The application of GB03 in combination with plant defense activators increased the efficacy of either GB03 or each activator in controlling blossom and shoot blight over two growing seasons.

In addition to agricultural and horticultural crops, GB03 was also evaluated in *Arabidopsis*. Results showed that *Arabidopsis* plants exposed to GB03 volatiles before pathogen inoculation exhibited a significantly lower severity of soft rot, caused by the bacterial pathogen *Erwinia carotovora* subsp. *carotovora*, compared with seedlings not exposed to GB03 volatiles (Ryu et al., 2004). Exogenous application of a racemic mixture of 2,3-butanediol isomers (RR and SS) triggered ISR. Ryu and colleagues reported that a biopreparation of GB03, IN937a formulated with the carrier chitosan also induced resistance against *P. syringae* pv. *tomato* in *Arabidopsis* (Ryu et al., 2007).

Taken together, GB03 has demonstrated broad-spectrum effectiveness against bacterial phytopathogens in various agricultural settings and plant species.

#### 3.2.3 Biological control of nematodes

Root-knot nematodes (*Meloidogyne* spp.) comprise one of the most economically damaging genera of plant parasitic nematodes affecting horticultural and field crops. GB03 has primarily been evaluated in formulations with other PGPR, using chitosan as the carrier, to assess its effectiveness in controlling root-knot nematodes. In field trials conducted in Florida, the PGPR formulation LS261 (*B. velezensis* GB03 and *B. cereus* C4) reduced the number of naturally occurring root-knot nematode galls on pepper plants compared to the control (Kokalis-Burelle et al., 2002). In another field study, the LS254 formulation (*B. velezensis* GB03 and *B. pumilus* SE34) significantly reduced the severity of galls formed by the root-knot nematode *Meloidogyne incognita* on muskmelon plants compared to the control group (Kokalis-Burelle et al., 2003). In field trials conducted in in Alabama, USA, *B. velezensis* GB03 alone or in combination with *B. amyloliquefaciens* IN937a or *B. subtilis* IN937b formulated in chitosan significantly suppressed the root-knot index in tomato compared to the control group (Kloepper et al., 2004a). However, the effectiveness of these formulations in reducing nematodes varies. In addition, GB03 failed to reduce the root-knot index in a field trial in Florida. GB03 with IN937a mixed in chitosan significantly suppressed the nematode index compared to the control. Overall, GB03, in combination with other PGPRs, has demonstrated notable effectiveness in controlling root-knot nematodes across various field trials.

#### 3.2.4 ISR against plant viruses and insect pests

Many studies have been conducted to investigate the ability of PGPR to suppress plant diseases caused by viruses and insects (Manjunatha et al., 2022). However, there is limited information available on the effectiveness of GB03 against plant viruses and insects. This may be partially because GB03, a model PGPR strain, has been tested, either alone or in combination with other strains, primarily for its potential to increase plant yield in various transplant systems (Kloepper et al., 2004a). Additionally, GB03-mediated plant protection against viruses and insect pests has been reported, not through direct antagonism, but through the indirect activation of ISR.

In the greenhouse and field trials, seed treatment with GB03 significantly reduced the incidence of *Bean common mosaic virus* (BCMV) on cowpea by 41% and 34%, respectively, compared to the untreated control (Shankar et al., 2009). Because combinations of multiple strains can be more effective than individual strains in controlling plant viruses, further studies were conducted to test the effect of GB03 in combination with other PGPR strains. A combination of GB03 with other PGPR strains significantly improved the control of BCMV on cowpea compared to individual strains (Shankar et al., 2009). The combination of GB03 and *B. pumilus* T4 was the most effective in reducing disease incidence compared to other combinations involving GB03.

In greenhouses, Ryu and co-workers evaluated a biopreparation (BioYield) of the PGPR *B. velezensis* GB03 and *B. amyloliquefaciens* IN937a formulated in chitosan as a carrier for its capacity to trigger ISR against viral diseases including CMV in *Arabidopsis* (Ryu et al., 2007). The biopreparation significantly reduced the severity of CMV compared to the untreated control. Similar results were obtained in tomatoes against CMV. Murphy et al. (2003) evaluated combinations of GB03 with one of the other PGPR strains formulated with the carrier chitosan for their ability to induce resistance in tomatoes against CMV. The authors reported that treating tomato plants with BioYield resulted in significantly lower CMV severity compared to the water control.

Some studies have shown that PGPR strains trigger ISR against not only plant pathogens but also insect pests that have a negative impact on crop yield (Kloepper et al., 2004b; Pieterse et al., 2014). However, there are fewer reports available on the effectiveness of GB03 against insects. *Arabidopsis* plants exposed to GB03 showed an increase in glucosinolate content and lower damage caused by the generalist herbivore, beet armyworm (*Spodoptera exigua*), compared to the water control (Aziz et al., 2016). GB03, when used as a seed treatment, has been shown to provide leaf protection against the specialist herbivore, diamondback moth (*Plutella xylostella*), on arugula, which is a close relative of *Arabidopsis* (Dos Santos et al., 2021). The induction of herbivore protection by GB03 was found to be correlated with greater induction of genes encoding glucosinolate biosynthesis enzymes.

A field study was conducted to investigate the potential of volatiles in insect management in cucumbers (Song and Ryu, 2013). In field trials conducted in South Korea, the effectiveness of two volatile organic compounds (VOCs), 3-pentanol and 2-butanone, was evaluated through soil drench application. These VOCs reduced bacterial angular leaf spot and decreased the population of aphid (*Myzus persicae*) nymphs and adults. Additionally, the number of ladybird beetles (*Coccinella septempunctata*), which are natural predators of aphids, showed a significant increase in the cucumber plants that were treated, as compared to the control group. GB03 has been reported to release VOCs including 2-butanone (Ryu et al., 2003). Thus, GB03 could be effective in controlling insects such as aphids. This warrants further investigation of its potential application in pest management in agricultural and horticultural production.

Increasing the uptake of insecticides in plants receiving the GB03 treatment could be beneficial for integrated pest management. GB03, when applied alone or in combination with *B. amyloliquefaciens* FZB24 was found to enhance the uptake of a neonicotinoid insecticide, thiamethoxam, in corn seedlings compared to the control (no bacterial treatment) (Myresiotis et al., 2015). Results revealed that the uptake and/or systemic translocation of thiamethoxam in the above-ground parts of corn plants, inoculated with GB03 and *B. amyloliquefaciens* FZB24, either individually or in combination, was significantly higher than in the untreated control. Thiamethoxam belongs to a relatively new class of insecticides known as neonicotinoids. It is registered for use on many crops to control a wide range of sucking and chewing insects, including aphids, beetles, thrips, whiteflies, and certain species of lepidopterans. GB03-elicited enhanced uptake of thiamethoxam could improve the efficiency of such insecticides at lower doses, serving as an alternative crop protection strategy.

In tomatoes, GB03 stimulated the systemic translocation of ASM, a wide-spectrum SAR inducer used in many crops to combat multiple diseases (Myresiotis et al., 2014). Treatment with the PGPR *B. velezensis* GB03 and *B. pumilus* SE34 significantly increased the absoption of ASM and its metabolites by the roots and their subsequent movement throughout the above-ground plant tissues, as compared to the control plants that were not treated. These studies indicated that PGPR application could help reduce the use of pesticides and improve the efficacy of SAR inducers in crop production. PGPR strains that enhance the uptake of agrochemical pesticides and SAR inducers by crops should be further investigated as an important component in integrated disease and pest management systems.

## 4 Induced systemic tolerance against abiotic stresses

In this section, we summarize the effects of GB03 on plant tolerance to high salinity and drought stress, as well as on plant nutrient homeostasis under various conditions (**Table 3**).

**Table 3:** Impact of GB03 on plant resilience to abiotic stress.

Treatments	Effect on plants	*In planta* mediators	Plant species	References
Entire GB03 VOC blend	Salt stress tolerance	HKT1; SOS3	*Arabidopsis thaliana*	Zhang et al., 2008a
GB03 soil inoculation	Salt stress tolerance	Not reported	*Trifolium repens* L.	Han et al., 2014
Entire GB03 VOC blend	Osmotic stress tolerance	PEAMT; glycine betaine; choline	*A. thaliana*	Zhang et al., 2010
GB03 soil inoculation	Drought stress tolerance	Not reported	*Lolium perenne* L.	Su et al., 2017
2,3-butanediol	Drought stress tolerance	NO signaling	*A. thaliana*	Cho et al., 2008
Diacetyl	Chlorophyll protection from low light, salt stress, or ABA	Antagonizing ABA effects	*A. thaliana*	Singh et al., 2022a
Entire GB03 VOC blend	Improved sulfur acquisition	ATP sulfurylase, APRs, APKs	*A. thaliana*	Aziz et al., 2016
Entire GB03 VOC blend	Improved iron acquisition	FIT1, IRT1, FRO2, root proton exudation	*A. thaliana*	Zhang et al., 2009
GB03 soil inoculation	Improved iron acquisition	Not reported	*Manihot esculenta*	Freitas et al., 2015
GB03 soil inoculation	Improved tolerance to nitrogen deficiency	Differential regulation of auxin and ABA	*Festuca arundinacea*	Wang et al., 2022
Diacetyl	Increased responses to phosphate deficiency	SA- and JA-mediated defense, GA signaling	*A. thaliana*	Morcillo et al., 2020a; Morcillo et al., 2020b

In *Arabidopsis* plants, GB03 VOCs significantly restored the biomass production induced by salt stress and simultaneously alleviated Na+ accumulation (Zhang et al., 2008a). GB03-induced increase in plant salt tolerance was also observed in white clover, which displayed significantly decreased shoot and root Na^+^ accumulation, leading to an improved K^+^/Na^+^ ratio (Han et al., 2014). The GB03 VOC-enhanced plant salt tolerance involves Na^+^ recirculation mediated by HKT1, which is responsible for Na^+^ exclusion from leaves by removing Na^+^ from the xylem sap, and Na^+^ exudation regulated by SOS3, which is required for the post-transcriptional activation of the H^+/^Na^+^ antiporter SOS1 that controls root Na^+^ exudation and long-distance Na^+^ transport in plants (Liu and Zhang, 2015; Zhang et al., 2020). Salt stress imposes both ionic and osmotic stresses in plant cells. In soybean plants, VOCs emitted from a *Pseudomonas simiae* strain increased salt tolerance by increasing the accumulation of osmoprotectant metabolites (Vaishnav et al., 2015). This protective mechanism probably also contributed to the enhanced salt tolerance in *Arabidopsis* by VOCs of GB03. The VOCs increased the accumulation of choline and glycine betaine, which are important osmoprotectants in plants under osmotic stress. As a result, the tolerance to drought stress was increased (Zhang et al., 2010). The addition of GB03, along with a water-retaining agent, significantly enhanced the protection against drought stress in perennial ryegrass (Su et al., 2017). The GB03 VOC 2,3-butanediol was shown to induce drought tolerance in a nitric oxide (NO) signaling-dependent manner (Cho et al., 2008). In addition, *Arabidopsis* and *Festuca arundinace* plants treated with GB03 VOCs via foliar spray or soil inoculation displayed reduced levels of abscisic acid (ABA) (Zhang et al., 2008a; Wang et al., 2022), a phytohormone that plays a central role in mediating plant resistance to various abiotic stresses (Zhang et al., 2022). The reduction in ABA levels, which indicates lower stress levels, was found to be correlated with the plant growth-promotion induced by GB03 under non-stress or nitrogen-deficient conditions (Zhang et al., 2008a; Wang et al., 2022). It should be noted that the reduced ABA levels in the stressed plants may also be a result of the GB03-mediated protection, rather than the cause of the protection. Similarly, diacetyl, another GB03 VOC, was recently shown to protect plants from premature senescence caused by low light intensity, high salinity, and exogenous ABA (Singh et al., 2022a).

GB03 displayed beneficial effects on plants under different nutrient conditions. In *F. arundinace* plants grown under nitrogen-deficient conditions, GB03 improved plant growth, which could be attributed, at least partially, to the substantial increase in auxin accumulation and decrease in ABA accumulation (Wang et al., 2022). This is significant because auxin is known to promote the development of lateral roots, which can enhance plant nutrient acquisition. While the exact role of auxin in facilitating plant stress resistance remains elusive, it is noteworthy that VOCs produced by certain fungal species have also been reported to induce plant tolerance to salt stress in a manner that depends on auxin. This is evident from the fact that the auxin signaling mutants *aux1-7, tir1-1*, and *axr1-3* did not exhibit increases in leaf surface area and lateral root density when exposed to fungal VOCs (Li and Kang, 2018). In Arabidopsis and cassava (*Manihot esculenta*), GB03 activates the iron acquisition machinery of plants, resulting in elevated endogenous Fe levels that support the higher amounts of the Fe-rich photosynthetic apparatus (Zhang et al., 2009; Freitas et al., 2015). The augmentation of photosynthesis trigerred by GB03 was also attributed to the enhanced acquisition of sulfur, another micronutrient important for photosynthesis. This was achieved through the transcriptional up-regulation of genes involved in the sulfur assimilation pathway (Aziz et al., 2016). Nonetheless, these studies investigated plant growth promotion under sufficient Fe and S conditions. However, it is unclear whether GB03 VOCs can enhance Fe and S acquisition in plants under nutrient-deficient conditions. GB03 VOCs displayed plant growth-promoting effects in plants grown in a phosphate (Pi)-sufficient medium. Additionally, these VOCs strongly enhanced plant Pi starvation responses (PSRs) in plants grown in a Pi-depleted medium, resulting in worsened PSRs compared to the untreated counterparts (Morcillo et al., 2020a; Morcillo et al., 2020b; Singh et al., 2022b). This interesting phenomenon was attributed to the VOC diacetyl. Together with another study on the transition from mutualism to incompatibility (Yang et al., 2022), it has led to the discovery of a new research area known as plant latent defense response (LDR), which is conditionally activated by certain non-pathogenic microbial factors and thus guards against potential risks from beneficial or commensal microbes (Zhang, 2023). This hidden layer of defense increases the complexity in comprehending PGPR-mediated plant nutrient uptake, owing to the crosstalk between immunity and nutrient-related processes. Additionally, activated immunity can alter root morphology, root exudation, and root microbiome (Morcillo et al., 2020a; Morcillo et al., 2020b; Singh et al., 2022b).

## 5 Modification of plant physiology and rhizosphere microbiome

Plants are naturally inhabited by a variety of epiphytic and endophytic microbes, which are collectively referred to as the plant microbiome. The assembly of the plant microbiome is shaped by the host via an integrated regulation of morphological structures, the exudation of secondary metabolites, and the innate immune responses (Kaushal et al., 2021; He et al., 2022; Lv et al., 2022; Yang et al., 2022). These factors are commonly influenced by PGPR-enhanced plant growth promotion and disease resistance. For instance, GB03 induces morphological changes in both the aerial parts and roots of plants, such as the enhanced expansion of leaf cells and enhanced production of adventitious roots in *Arabidopsis* and *Brachypodium distachyon* (L.) Beauv (Zhang et al., 2007; Delaplace et al., 2015). As a biocontrol agent, GB03 was shown to be superior to three other examined commercial biocontrol strains in suppressing the *R. solani* infection in potato field experiments (Larkin, 2016). In addition to influencing the plant, soil microbes also contribute to the shaping of the rhizosphere microbiome through interactions with other microbes in the same microenvironment. For instance, the colonization of *Arabidopsis* roots by GB03 in a hydroponic system was increased by one of the three helper strains, including *Agrobacterium* sp. ES981, *Variovorax* sp. ES1063, and *Methylobacterium* sp. ES1084 (Eckshtain-Levi et al., 2020).

The effects of inoculating field-grown potatoes with GB03 on their root microbiome were evaluated using two methods: Single Carbon Source Substrate Utilization (SU) and Soil Fatty Acid Methyl Ester (FAME) profiles. Generally, the GB03-treated potatoes exhibited increased microbial activity and improved SU (Larkin, 2016). Recently, the impacts of GB03 inoculation on the root microbiome in tomato plants were directly shown through 16S rRNA gene profiling (Kong et al., 2021). The microbiome of GB03-treated plants was characterized by an approximately 20% increase in bacilli abundance and a 15% reduction in Gammaproteobacteria abundance. While the increase in bacilli could be due to the inoculation of GB03, the changes in Gammaproteobacteria abundance were attributed to the GB03-mediated regulation of plant physiology. This pattern was not observed in GB03-inoculated soil without plants (Kong et al., 2021). Importantly, leaves of GB03-treated tomato plants released β-caryophyllene as a signature VOC, which elicited the release of a significant amount of salicylic acid (SA) in the root exudates of nearby tomato seedling. As a result, the root microbiome composition in neighboring plants became synchronized (Kong et al., 2021).

## 6 Antimicrobial compound production in *B. velenzensis* GB03

Antibiotic biosynthetic gene clusters (BGCs) are specific groups of genes that work together to produce antibiotics. These clusters are essential for the synthesis of antimicrobial compounds that help organisms defend against bacterial infections. Analysis of *B. velezensis* GB03 genome revealed 10 BGCs that produce three lipopeptides (surfactin, bacillomycin D, and fengycin), one siderophore (bacillibactin), three polyketides (macrolactin, bacillaene, and difficidin), two bacteriocins (mersacidin and amylocylicin), and one dipeptide (bacilysin) with antibacterial properties (**Table 4**). The lipopeptides, polyketides, and siderophore are synthesized via a 4′-phosphopantetheinyl transferase (Sfp)-dependent nonribosomal mechanism (Quadri et al., 1998). Bacteriocins are small peptides synthesized by the ribosome (Benítez-Chao et al., 2021). The dipeptide is produced by an Sfp-independent nonribosomal pathway (Islam et al., 2022). The BGCs are highly conserved in the *B. amyloliquefaciens* group, including *B. amyloliquefaciens*, *B. velezensis*, and *B. siamensis* (Fan et al., 2017). The ability of GB03 to produce various antibiotics makes it a powerful weapon against plant pathogens.

**Table 4:** Antibiotics produced by *B. velezensis* GB03 and their biosynthetic gene cluster.

Antibiotics	Compounds	Gene structure^a^
Lipopeptide	Surfactin	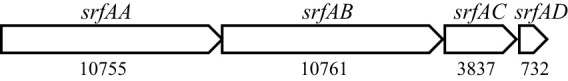
	Fengycin	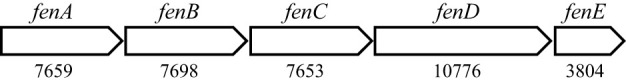
	Bacillomycin D	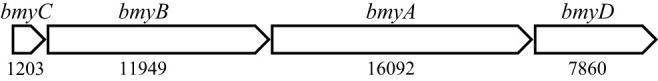
Siderophore	Bacillibactin	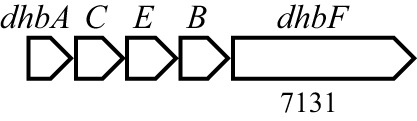
Polyketide	Bacillaene	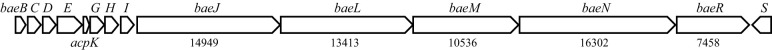
	Difficidin	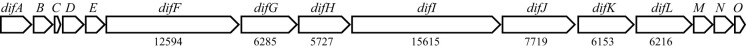
	Macrolactin	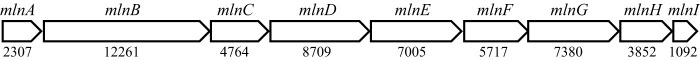
Bacteriocin	Mersacidin	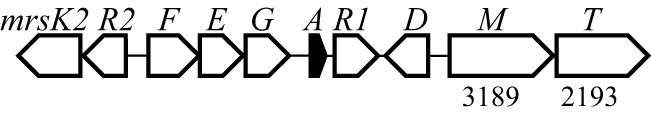
	Amylocyclicin	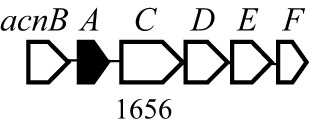
Dipeptide	Bacilysin	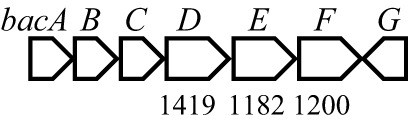

a The numbers indicate gene size in base pairs. The closed boxes are core genes of the bacteriocins

### 6.1 Lipopeptides

Surfactin is an amphiphilic compound composed of a cyclic heptapeptide linked to an N-terminal β-hydroxy fatty acid. The BGC for surfactin in the GB03 genome consists of four open reading frames (ORFs) identical to those previously reported (Théatre et al., 2021). It has been reported that surfactins have relatively low, but broad, antibacterial activities against both Gram-positive and -negative bacteria (Ndlovu et al., 2017; Lilge et al., 2022). In addition, they have antiviral, anticancer, antimycoplasma, antihypercholesterolemia, and anti-inflammatory properties, as well as anti-adhesion activity against pathogens (Chen et al., 2022). Therefore, surfactins have the potential to be developed as therapeutic agents.

Fengycin is a decapeptide linked to the N-terminal β-hydroxy fatty acid (Vanittanakom et al., 1986). Fengycin BGC in the GB03 genome consists of five ORFs including fenA, fenB, fenC, fenD, and fenE. It has been reported that fengycins exhibit antitumor (Cheng et al., 2016) and antiviral (Kang et al., 2021) activity, as well as strong and broad-spectrum antifungal activity against phytopathogenic fungi (Guo et al., 2014; Fan et al., 2017). In addition, an analysis of the effect of gut microbiome composition on *Staphylococcus aureus* colonization in 200 healthy individuals demonstrated that fengycin produced by gut *Bacillus* species could eliminate *S. aureus* from the human body through signal interference (Piewngam et al., 2018). The results suggest that GB03 has the potential to be used as a therapeutic probiotic, as well as an agricultural agent for plant protection.

Bacillomycin D (BD) is a cyclic antifungal lipopeptide, consisting of seven amino acids and an N-terminal hydrophobic fatty acid chain. The BD BGC in the GB03 genome is composed of four ORFs (bmyC, bmyB, bmyA, and bmyD). Genetic studies of *B. velezensis* FZB42 showed that the antifungal activity of the *fenA*-deficient mutant was similar to that of the wild type. However, the *bmyA* mutant showed less antifungal activity, while the *fenA-bmyA* double mutant showed no antifungal activity (Koumoutsi et al., 2004). These results indicate that both BD and fengycin contribute to the antifungal activity of *B. velezensis* species. Additionally, BD has been reported to exhibit antioxidant (Tabbene et al., 2012) and anticancer (Lin et al., 2019) activities, suggesting that BD also has potential as a therapeutic agent like surfactin and fengycin.

### 6.2 Siderophore

The GB03 genome includes a bacillibactin BGC. Bacillibactin is a catechol-based siderophore, which is a high-affinity Fe-chelating molecule. Since Fe is essential for all microorganisms, siderophores can suppress pathogens by competing for Fe uptake (Khasheii et al., 2021). Bacillibactin consists of three 2,3-dihydroxybenzoate (DHB) groups attached to a cyclic amino acid core and is synthesized by NRPSs encoded by the *dhb* operon (May et al., 2001). According to a recent study, bacillibactin has a direct antibiosis activity, rather than Fe scavenging activity, against nonsusceptible bacterial and fungal phytopathogens (Dimopoulou et al., 2021).

### 6.3 Polyketides

Bacillaene, a linear polyketide antibiotic, is synthesized non-ribosomally by a mega-sized (approximately 2.5 MDa) polyketide synthase (PKS) complex (Butcher et al., 2007). The BGC for bacillaene in the GB03 genome consists of 5 *PKSs* and 11 auxiliary genes. Bacillaenes are considered to play an important competitive role in *Bacillus* survival because they exhibit broad antifungal activity against a variety of plant pathogens, as well as broad-spectrum antibacterial activity against both Gram-negative and -positive bacteria (Miao et al., 2023). However, the highly unstable nature of bacillaenes hinders their identification, characterization, and application (Li et al., 2019).

Difficidin is a highly unsaturated macrocyclic polyene lactone phosphate ester belonging to a family of 22 polyketides. It exhibits antibiotic properties and is sensitive to low pH, high temperature, and oxidation (Wilson et al., 1987). Like bacillaene, difficidin is also synthesized non-ribosomally by a large PKS complex (Chakraborty et al., 2021). The BGC for difficidin in the GB03 genome consists of 15 *PKSs* and auxiliary genes. Difficidins exhibit broad-spectrum antibacterial activity against Gram-negative and -positive bacteria (Wilson et al., 1987). A recent study reported the antibacterial activity of difficidin against vancomycin-resistant *Enterococcus faecalis*, methicillin-resistant *S. aureus*, and other drug-resistant bacteria such as *Klebsiella pneumoniae* and *Pseudomonas aeruginosa* (Chakraborty et al., 2021). These results show that difficidin has the potential to be used as an antibiotic for treating multidrug-resistant bacterial pathogens.

Macrolactin, synthesized by a PKS complex, is a macrolide that contains three distinct diene structural elements within a 24-membered lactone ring (Ortiz and Sansinenea, 2020). The BGC for macrolactin in the GB03 genome consists of nine genes. *B. amyloliquefaciens, B. velezensis*, and *B. siamensis* form an operational group because they are closely clustered in the phylogenetic tree. However, macrolactin is found only in *B. velezensis* among the three species (Fan et al., 2017). Macrolactins exhibit not only potent antibacterial activity against Gram-negative and -positive bacteria but also antifungal, anti-inflammatory, anticancer, and antiviral activities (Ortiz and Sansinenea, 2020). Therefore, macrolactin has great potential for various applications in the agricultural and pharmaceutical fields.

### 6.4 Bacteriocin

Mersacidin is a ribosomally synthesized and post-translationally modified lanthipeptide with unique intramolecular structures, including a very small lanthionine (Viel and Kuipers, 2022). Mersacidin modification requires the extracellular protease AprE, in addition to the mersacidin BGC products (Viel and Kuipers, 2022). Mersacidin BGC in the GB03 genome consists of 10 ORFs for structural gene, maturation, transporter, immunity, and regulator. It has been reported that mersacidin exhibits antimicrobial activity against Gram-positive bacteria and inhibits peptidoglycan synthesis by targeting lipid II (Brötz et al., 1998).

Amylocyclicin is a circular, highly hydrophobic bacteriocin synthesized by ribosomes. Amylocyclicin has been reported high antibacterial activity against closely-related Gram-positive bacteria, but not against Gram-negative bacteria. Amylocyclicin BGC in the GB03 genome consists of six ORFs for structural genes, maturation, transporters, and immunity.

### 6.5 Dipeptide antibiotic

Bacilysin is a dipeptide antibiotic composed of N-terminal L-alanine and C-terminal L-anticapsin. The bacillicin BGC in the GB03 genome contains seven ORFs that encode enzymes responsible for the nonribosomal biosynthesis of bacillicin. Bacilysin has been reported to possess broad-spectrum antimicrobial activity against bacteria, fungi, and algae. Its activity is primarily depent on the C-terminal L-anticapsin, which inhibits glucosamine 6-phosphate synthase and prevents the formation of microbial cell walls (Islam et al., 2022). Bacilysin is stable at high temperatures (100°C for 15 mins) and a wide pH range (1.4-12.0), making it ideal for applications in the pharmaceutical industry, as well as in the food and agriculture sectors (Özcengiz and Alaeddinoglu, 1991).

## 7 VOC emission from *B. velenzensis* GB03

We prefer to use a term bacterial volatile compounds (BVCs) rather than VOCs because certain bacteria like *Pseudomonas* spp. produce non-organic volatile compounds. However, GB03 did not report non-organic compounds previously. VOCs are organic chemicals that readily evaporate into the air. In the context of plant-microbe interactions, VOCs play a crucial role as signaling molecules that facilitate communication between plants and beneficial microbes, thereby enhancing plant growth and resistance to pathogens. *B. velezensis* GB03 produces a blend of VOCs, which greatly influence plant physiology. Remarkably, GB03 promotes plant growth by emitting a cocktail of VOCs without the need for direct physical contact (Ryu et al., 2003). The pioneering study that explored the VOCs released by GB03 revealed that this PGPR strain produces a greater quantity and a wider range of VOCs compared to other non-plant growth-promoting bacteria (Ryu et al., 2003).

Among the VOCs produced by *B. velezensis* GB03, a substantial amount is accounted for by acetoin and 2,3-butanediol. These VOCs significantly contribute to the growth of *Arabidopsis* plants (Ryu et al., 2003). In addition, these compounds enable long-distance communication among *Arabidopsis* seedlings, which in turn defend the plants from invasion by the phytopathogen *Pectobacterium carotovorum* subsp. *carotovorum* through the induction of ethylene-dependent ISR (Ryu et al., 2004).

The VOCs emitted by GB03 also confer other physiological advantages to plants, including improved resistance against abiotic stresses like salinity, drought, and osmotic imbalance, as well as increased photosynthetic efficiency (Zhang et al., 2008a; Zhang et al., 2010). Moreover, when plants recognize the VOCs emitted by GB03, the genes associated with sulfur assimilation are activated, resulting in an increase in sulfur uptake and accumulation in *A. thaliana*. This, in turn, elevates the concentration of glucosinolates in the plant, providing a defense mechanism against *Spodoptera exigua* (Abdel-Aziz et al., 2023). Furthermore, certain VOCs produced by GB03, such as glyoxylic acid, 3-methyl-butanoic acid, and diethyl acetic acid, are categorized as organic acids and can acidify the rhizosphere. This process enhances Fe mobility in the rhizosphere, promoting better Fe absorption by plants (Zhang et al., 2009).

These VOCs were identified through the cultivation of *B. velezensis* GB03 on artificial media (Farag et al., 2017). However, a more recent study revealed the presence of 2-nonanone among the VOCs, a compound not previously detected in GB03 (Riu et al., 2022a; Riu et al., 2022b). This compound was found in both soil and on artificial agar media, indicating its potential for natural production in agricultural fields. Notably, 2-nonanone released by GB03 activates ISR in tomato plants, offering them protection against *P. syringae* pv. *tomato* even at extremely low concentrations like nM levels (Riu et al., 2022a). This further underscores the significant role of GB03-emitted VOCs in promoting plant health and growth. Although their functions remain unclear, GB03 produces substantial amounts of non-major volatiles, such as 1-propanol-2-methyl, butanal-3-methyl, acetic acid diethyl, ethyl acetate, and isoprene, among others (Farag et al., 2006).

## 8 Formulation and commercialization

Bacilli including *Bacillus* spp. and *Paenibacillus* spp. could be one of the major sources of potential microbial commercial products (Ongena and Jacques, 2008) for the following reasons. First, the US Food and Drug Administration (USFDA) has granted the “generally regarded as safe” (GRAS) status to *B. subtilis* and related species, recognizing them as non-pathogenic (Harwood and Wipat, 1996). Second, bacilli have the capacity to produce endospores (Piggot and Hilbert, 2004), which are extremely resistant to and can remain dormant under unfavorable environmental stresses such as high temperature, low or high pH, nutrient deficiency, and drought. This allows for the commercialization of bacilli for long-term usage, similar to synthetic agrochemicals and fertilizers (Monteiro et al., 2005). Third, *B. subtilis* is a well-studied model microorganism and, like *Escherichia coli*, can be genetically modified. Bacilli-related information accumulated over the long term contributes to a better understanding of their physiology, facilitates their mass production, and helps in generating commercial formulations. Below, we present examples of commercial products generated from *B. velezensis* GB03. In 2008, the USFDA ruled that GB03 was exempt from inducing tolerance in plants, as stated: “The biofungicide *B. velezensis* GB03 is exempt from the requirement of a tolerance in or on all raw agricultural commodities when used in accordance with good agricultural practices [73 FR 50556, Aug. 27, 2008]”.

**Kodiak^®^**: *B. velezensis* GB03 is commercially registered in the USA as a biocontrol and growth-promoting product, named Kodiak^®^ (Gustafson, Inc., Plano, TX, USA), which is considered as one of the most effective biological control products currently available in the market. The use of Kodiak^®^ is widespread and versatile. It is commonly applied in synergy with conventional fungicides a seed treatment to bolster crop growth. Kodiak^®^, a concentrated formulation of GB03 spores, is applied directly to plant seeds with the primary intention of controlling soil-borne phytopathogens. Though GB03 is widely recognized for its inherent ability to efficiently suppress notorious phytopathogens such as *Fusarium* spp. and *R. solani* (Brannen and Backman, 1994; Zhang and Howell, 1995), the application of Kodiak^®^ in conjunction with standard chemical fungicides amplifies the defensive capabilities of plants. This powerful combination provides robust protection against phytopathogen infection, consequently leading to improved crop yield (Brannen and Kenney, 1997). Another noteworthy aspect of *B. velezensis* strain GB03 is its ability to colonize the rhizosphere of cotton plants. Thus, Kodiak^®^ offers long-term active protection in combination with fungicides. This treatment not only controls pathogens in the early seedling stages but also effectively suppresses chronic and subacute diseases in cotton plants (Brannen and Kenney, 1997).

**Companion^®^**: Like Kodiak^®^, Companion^®^ (Advance Grass Solution Ltd., UK) is a commercial plant health product formulated from *B. velezensis* strain GB03, and is widely used in horticultural and agricultural production (Kloepper et al., 2004a; Anckaert et al., 2021). Companion^®^ application facilitates the rapid colonization of plant roots by GB03, leading to enhanced growth and effective pathogen inhibition across a variety of plant species (Anckaert et al., 2021). Companion^®^ was developed to manage infections caused by *Sclerotinia, Rhizoctonia, Fusarium*, and *Aspergillus* in pod vegetables, tomatoes, cotton, peanuts, soybeans, wheat, barley, corn, strawberries, and grapes (Anckaert et al., 2021).

**BioYield™**: The successful commercialization and market acceptance of Kodiak^®^ has served as a catalyst for further research and development in the field of biocontrol agents and fertilizers using *Bacillus* species. One notable example is Yield Shield (Bayer CropScience, USA), which consists of the spores of the endophytic bacterium *B. pumilus* strain INR7 (or GB34). BioYield™ was registered by the United States Environmental Protection Agency (EPA) in 2003, and has been used extensively for the biocontrol of insect pests in an array of crops including legumes, cereals, vegetables, sugar beet, and cotton (Miljakovic et al., 2020). Expanding on the synergistic effects of *B. velezensis* GB03, several products have been developed that combine two strains of *Bacillus* species. BioYield™ (Gustafson, Inc., Plano, TX, USA) contains the endophyte *B. amyloliquefaciens* IN937a along with the non-endophyte *B. velezensis* GB03 (Kloepper et al., 2004a). BioYield™ consists of various components that employ different mechanisms to control crop diseases (Kloepper et al., 2004a). In BioYield™, GB03 was included to control of soil-borne pathogens by producing the antibiotic iturin. *B. amyloliquefaciens* IN937a was included for its ISR against crop pathogens. Chitosan was used to control nematodes by promoting the growth of indigenous soil antagonists that target root-knot nematodes. When combined with GB03, Yield Shield significantly mitigated the severity of CMV under greenhouse conditions (Murphy et al., 2003).

The regulatory approval process for microbial products, as described above, involves rigorous assessments of safety and efficacy. A comprehensive dossier, including laboratory and field trial data, is submitted to relevant regulatory bodies for scientific review and risk evaluation. Upon successful review, the product is registered and subjected to ongoing monitoring to ensure that it meets safety and efficacy standards. Thus, these products are deemed safe for use.

## 9 Plant quality improvement

The application of PGPR and synthetic microbial communities has been recently considered by many breeders, nutritionists, and farmers for plant quality improvement (Etalo et al., 2018; Cellini et al., 2021). Plant quality is mostly determined by the presence of secondary metabolites, which iclude flavor compounds like essential oils and aromas. There is a strong connection between the induction of plant resistance and an increase in crop value. In 2009, exposure of sweet basil plants to GB03 VOCs increased the amounts of two major essential soil compounds, alpha-terpineol and eugenol, by ca. 2- and 10-fold, respectively (Banchio et al., 2009). In addition to basil, GB03 volatiles also promoted the essential oil contents of two medicinal plant species, *Codonopsis pilosula* and *Atractylodes lancea* (Wu et al., 2016; Zhou et al., 2016). In the model plant Arabidopsis, strain GB03 modified photosynthesis by alleviating the negative feedback regulation of sugar accumulation (Zhang et al., 2008b). *Arabidopsis* HEXOSE SENSOR KINASE 1 (AtHXK1) detects hexose concentrations after its accumulation during photosynthesis. AtHXK1 negatively regulates photosynthesis at high sugar contents (Cho et al., 2010). *Arabidopsis* treated with GB03 BVCs promoted the accumulation of hexose sugars (Zhang et al., 2008b).

In addition to secondary metabolites, the mineral contents of plants also influence animal and human health. Increased Fe uptake is vital for plants as iron, a crucial component of chlorophyll, is essential for the photosynthesis process. *Arabidopsis* treated with GB03 increased Fe uptake by up to 2-fold (Zhang et al., 2009; Wang et al., 2017). Beyond *in vitro* experiments, similar results were also obtained in the greenhouse, where Fe accumulation and photosynthetic efficiency were increased in cassava plants exposed to GB03 (Freitas et al., 2015). The field application of strain GB03 increase total N and total P contents in tall fescue (Wang et al., 2022). Such results demonstrate that strain GB03 enhances the production of commercially valued secondary metabolites and minerals in herbaceous, medicinal, and crop plants.

## 10 Limitations

Here, we summarized previous studies on *B. velenzensis* GB03, which provide an important gateway for promoting plant health and growth. Since the early 1970s, many scientists used strain GB03 as a model bacterial inoculant for seeds, seedlings, and soilless mixture, and as a model for studying plant–rhizobacteria interactions and for searching bacterial determinants on plant growth promotion and induced systemic resistance (ISR). Recently, modern multi-omics technologies including genomics and metabolomics have allowed us to understand the nature of strain GB03, both *in situ* and in planta. Despite the successful application of strain GB03, certain limitations still remain.

### 10.1 Insect and nematode control

The effects of GB03 on promoting plant growth and controlling plant diseases caused by fungal, bacterial, and viral pathogens, as well as insect pests like cucumber beetles, have been reported in numerous studies. However, little is known about the efficacy of GB03 against plant-parasitic nematodes. More studies are needed to explore the potential of GB03 for controlling nematodes in agricultural and horticultural crops. Future studies could also focus on the effects of GB03 on reducing economically important insects by increasing their natural enemies. Additionally, research could explore the potential of GB03 to enhance the uptake and translocation of insecticides or resistance inducers in crops, thereby improving pest and disease management more effectively.

### 10.2 Finding bacterial determinants

While a variety of antimicrobial substances and VOCs that promote plant growth and/or trigger ISR in various plants have been identified and characterized, many other bioactive secondary metabolites produced by GB03 remain undiscovered. To uncover these novel molecules that positively influence plant physiology, further studies employing ‘multi-omics’ approaches are necessary.

### 10.3 Molecular mode of action

While GB03 has multiple beneficial effects on plants, the molecular mechanism underlying the phenotype remains largely elusive. For example, it is unclear how the VOCs integrally modulate different biological processes in stressed plants to achieve optimized outcomes: they may enhance stress tolerance by altering hormonal pathways, influence plant morphology by modulating growth regulators, and shape the microbiome by selectively promoting the growth of beneficial microbes.

### 10.4 Synthetic community-based bio-inoculants

The molecular basis of plant interaction with beneficial Gram-positve PGPR such as bacilli is largely unknown. In light of studies on Microbe-Induced Plant Volatiles (MIPV), understanding this concept is crucial for the application of GB03 in synthetic community-based bio-inoculants. These volatiles serve as a communication channel between plants and microbes, enhancing plant health while reducing the need for chemicals. The MIPV concept was initially introduced through research on the interaction between tomato roots and GB03. As such, we anticipate new findings on the nature of interactions between bacilli, plants, and their microbiomes in the future. As we mentioned previously, the spore-forming PGPR show profound potential for generating commercial products. Recent metagenome analyses demonstrated how strain GB03 orchestrated indigenous microbial community by modulation of plant physiology. By combining the molecular understanding of plant responses with the impact of indigenous microbiota on each target crop, the precision formulation based on GB03 will provide an alternative solution for problems that were previously unsolvable using tranditional methods. Additionally, the combination of synthetic agrochemicals (pesticides and fertilizers) with strain GB03 as an adjuvant can also be considered. GB03 can reduce the concentration and dosage of chemicals applied to crops.

## 11 Conclusions

Overall, we provide a brief history of a PGPR strain isolated from wheat roots in 1971 in Australia. Owing to the tremendous effort of many scientists, industry workers, and farmers, strain GB03 has become a model Gram-positive PGPR. Many other Gram-positive bacteria are following the avenue paved by strain GB03. While we have overcome many limitations over the years, we face new obstacles including climate change, food supply for the increasing human population, and the management of new and re-emerging pathogens. Biological inoculants prepared from GB03 are clear alternative solutions to improving plant and planetary health by reducing chemical usage and greenhouse gas emission, and increasing biotic and abiotic stress resistance for another 50 years.

## Author contributions

SJ: Visualization, Writing – original draft, Writing – review & editing. S-KC: Validation, Writing – original draft, Writing – review & editing. HZ: Funding acquisition, Validation, Writing – original draft, Writing – review & editing. SZ: Validation, Writing – original draft, Writing – review & editing. C-MR: Conceptualization, Funding acquisition, Project administration, Supervision, Validation, Writing – original draft, Writing – review & editing. JK: Conceptualization, Writing – original draft, Writing – review & editing.
